# Angioedema After Accidental Semaglutide Dosing Error: A Case Report

**DOI:** 10.3390/jcm15103705

**Published:** 2026-05-12

**Authors:** Bryan D. Kraft, Sarah Matuszak

**Affiliations:** 1 Division of Pulmonary and Critical Care Medicine, Washington University School of Medicine, 660 South Euclid Avenue, St. Louis, MO 63110-1010, USA; 2 Department of Pharmacy, Barnes-Jewish Hospital, St. Louis, MO 63110, USA

**Keywords:** glucagon-like peptide-1 receptor agonists, angioedema, drug-related side effects and adverse reactions, angiotensin receptor antagonists, drug compounding, semaglutide, case report

## Abstract

**Background:** Glucagon-like peptide-1 receptor agonist (GLP-1 RA) use has increased exponentially as studies show significant benefits in cardiovascular and renal diseases and obesity. Accessibility to the public also increased after compounding pharmacies began direct-to-consumer distribution. Gastrointestinal side effects are common; however, hypersensitivity reactions are rare. **Case Presentation:** A 50-year-old female with a history of obesity, hypertension, and lisinopril-induced angioedema presented to the Emergency Department with swelling of the lips, tongue, and throat developing four hours after her first injection of compounded semaglutide for weight loss. She was treated with epinephrine, corticosteroids, and antihistamines, but due to progressive airway edema, she required intubation and mechanical ventilation for four days. After extubation, she reported accidentally injecting a ten-fold higher dose (2 mg) of semaglutide than was appropriate for the first dose. The hospitalization was complicated by hypoglycemia requiring dextrose infusion, but was otherwise unremarkable, and she was discharged home on day 7. Based on the temporal onset after semaglutide injection, this presentation was most consistent with GLP-1 RA-induced angioedema. While she also had a history of lisinopril-induced angioedema five years earlier, and had been taking valsartan for hypertension, the remoteness of the lisinopril exposure made this less likely. **Conclusions:** Semaglutide use may be associated with severe angioedema within hours of administration. Given the overlapping indications and patient populations, angioedema appearing in patients taking both GLP-1 RAs and ACE inhibitors may become increasingly common and present a diagnostic dilemma. Diagnosis of hypersensitivity to GLP-1 RAs can be supported with history and positive skin testing. Clinicians should be aware that inexperienced patients are at the highest risk of dosing errors.

## 1. Introduction

The use of glucagon-like peptide-1 receptor agonists (GLP-1 RAs) has increased exponentially over recent years as accumulating data support broad benefits outside of glycemic control, including reducing cardiovascular disease [1,2], kidney disease [3,4], and obesity [5]. This surge, combined with accompanying drug shortages, has led online and compounding pharmacies to sell injectable GLP-1 RAs directly to consumers since 2022. However, reports of dosing errors and overdoses have emerged in patients who lack familiarity with injections [6]. This case illustrates a clinically instructive report of both a rare adverse drug effect (severe angioedema) arising hours after the first semaglutide injection, as well as a significant dosing error made by an inexperienced patient. The case also highlights the diagnostic dilemma arising when patients are taking more than one drug that can cause angioedema.

## 2. Case Presentation

**Figure 1:**
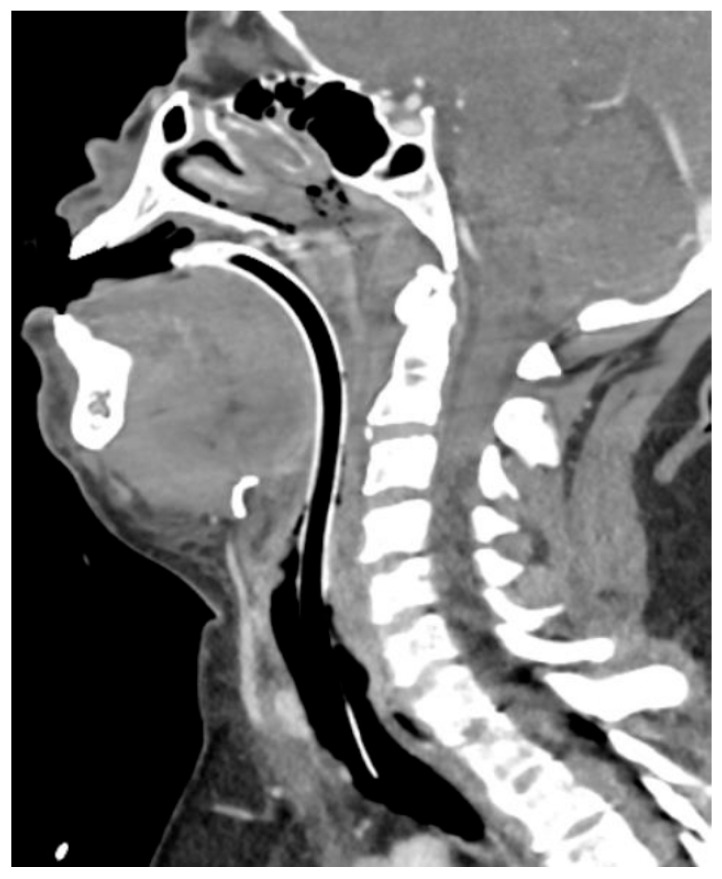
Sagittal image from a computed tomography scan of the neck with intravenous contrast, demonstrating a collapsed hypopharyngeal and laryngeal airway due to surrounding tissue edema, with endotracheal tube in situ securing the airway.

A 50-year-old female presented to the Emergency Department via ambulance after developing lip and tongue swelling starting approximately four hours after self-administering her first semaglutide injection. Her medical history included obesity (BMI 44), obstructive sleep apnea (on continuous positive airway pressure), moderate persistent asthma (budesonide–formoterol 160-4.5 mcg inhaler twice daily, montelukast 10 mg daily, and albuterol inhaler as needed), depression (bupropion XL 150 mg daily and sertraline 100 mg daily), hypertension (valsartan 80 mg daily), and lisinopril-induced angioedema five years earlier. The patient had been in her usual state of health prior to the reaction, and there was no history of preceding severe stress, respiratory infection, physician exertion, alcohol or drug use, or other new medications. En route to the hospital, she was given epinephrine 0.3 mg IM, methylprednisolone 125 mg IV, diphenhydramine 50 mg IV, and nebulized albuterol. Upon arrival at the Emergency Department, the patient’s vital signs were a temperature of 36.8 °C, a heart rate of 78, a respiratory rate of 22, a blood pressure of 167/85, and a pulse oximetry reading of 100% on room air. There was obvious lip, tongue, and neck swelling, although Emergency Medical Services personnel reported that the lip swelling had improved with treatment. Heart and lung exams were normal. There was no urticaria or other skin rashes observed. She was diagnosed with angioedema and treated with an additional dose of epinephrine 0.3 mg IM (Hospira, Inc. Lake Forest, IL, USA), famotidine 20 mg IV (Baxter Healthcare Corporation, Deerfield, IL, USA),, and tranexamic acid 1000 mg IV (Amneal Pharmaceuticals, Bridgewater, NJ, USA); however, approximately 30 min later, her lip and tongue swelling worsened, and the decision was made to proceed with endotracheal intubation for airway protection. The intubation was difficult due to profound glottic edema seen on video laryngoscopy, requiring rescue with a laryngeal mask airway (i-gel, Intersurgical, Indianapolis, IN, USA) and insertion of a 6.0 endotracheal tube (Shiley, Medtronic, Dublin, Ireland) on the second attempt. The patient was then transferred to our hospital for a higher level of care. The first venous blood gas after intubation reported a pH of 7.22, pCO_2_ of 51 mmHg, pO_2_ of 54 mmHg, and bicarbonate of 22 mmol/L. The acidosis improved on a subsequent venous blood gas drawn two hours later, which reported a pH of 7.34, pCO_2_ of 53 mmHg, and bicarbonate of 29 mmol/L. A computed tomography scan of the neck with intravenous contrast (Optiray-350 IV, Libel-Flarsheim, Raleigh, NC, USA) demonstrated diffuse supraglottic and glottic laryngeal edema with collapse of the hypopharyngeal and laryngeal airway, as well as edema within the submandibular and submental spaces, consistent with a diagnosis of angioedema, and ruled out a focal mass or fluid collection causing external compression of the larynx (Figure 1). Serum tryptase and complement C4 collected approximately 5 h after her initial presentation were normal. She was admitted to the intensive care unit for acute respiratory failure due to angioedema and treated with dexamethasone 10 mg IV q8 hours (Civica, Inc., Lehi, UT, USA), famotidine 20 mg IV q12 hours (Baxter Healthcare Corporation, Deerfield, IL, USA), and diphenhydramine 25 mg q6 hours (Hikma Pharmaceuticals USA Inc., Cherry Hill, NJ, USA).

**Figure 2:**
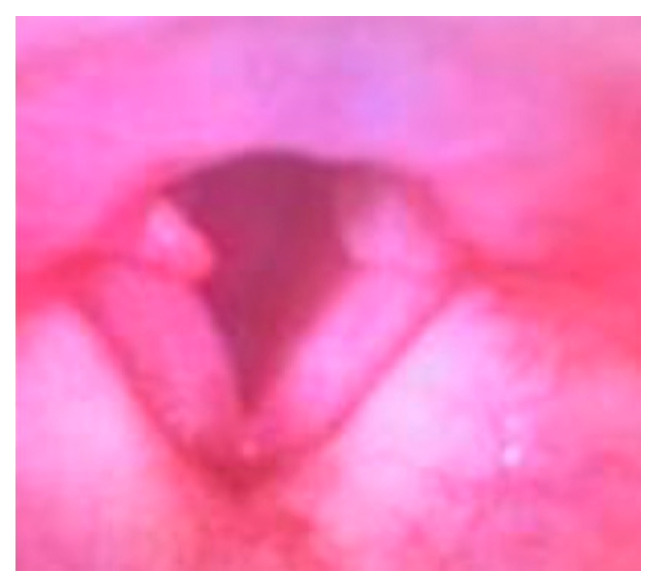
Image from flexible laryngoscopy of glottic structures after extubation showing resolution of edema and bilateral vocal process granulomas.

Over the next several days, the swelling of the lips and tongue improved. On hospital day 3, the patient developed an air leak around the endotracheal tube when the cuff was deflated, suggesting the laryngeal edema had improved. The steroids and histamine blockers were held; however, she was left intubated for one more day to ensure the edema was continuing to resolve and did not worsen again off therapy. On hospital day 4, after confirming an air leak was still present, she was extubated over an 8 French airway exchange catheter (Cook Medical, Bloomington, IN, USA), that was removed after several minutes. A follow-up flexible laryngoscopy soon after extubation showed complete resolution of the tongue and glottic edema, as well as intact function of the true vocal cords, and absence of mucosal or anatomical abnormalities (Figure 2). Bilateral vocal process granulomas were seen and felt likely due to the recent intubation.

After extubation, the patient provided additional history that she had purchased semaglutide from a popular direct-to-consumer online pharmacy. The medication was dispensed in a 2.5 mg/mL vial with instructions to inject 8 units (0.2 mg) using a U-100 insulin syringe; however, due to confusion about the units, she administered 80 units (2 mg), or 10× the intended dose for her first dose.

**Figure 3:**
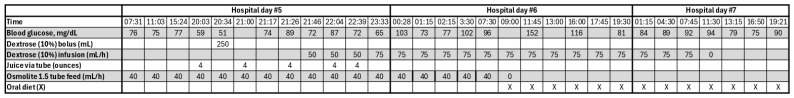
Timeline of development and treatment of hypoglycemia. “#” refers to hospital day number.

On hospital day 5, despite receiving enteral nutrition via nasogastric tube (Osmolite 1.5 at 40 mL/h, Abbott, Abbott Park, IL, USA), the patient exhibited hypoglycemia that was treated initially with enteral juice, but ultimately required a 10% dextrose bolus (250 mL, B Braun Medical Inc., Bethlehem, PA, USA) and a continuous intravenous infusion of 10% dextrose for approximately 36 h (starting at 50 mL/h and increasing to 75 mL/h) to maintain euglycemia (Figure 3). On hospital day 6, she began to eat food by mouth, though she had intermittent nausea and vomiting. The patient was transferred out of the intensive care unit later that day. The dextrose infusion was discontinued on hospital day 7. Blood glucose remained within the normal range thereafter, though her oral intake was reduced due to persistent nausea. On hospital day 8, she was discharged home. She was offered outpatient skin testing by allergy consultants to further investigate hypersensitivity to semaglutide, but she declined. She was advised to stop taking both valsartan and semaglutide.

## 3. Discussion

This case highlights a rare but serious suspected adverse drug reaction after injecting compounded semaglutide, accompanied by a significant dosing error. Angioedema due to GLP-1 RAs is an exceptionally rare but reported adverse drug reaction, with pooled estimates of 4.2 per 10,000 person-years [7]. Onset of symptoms has been reported to occur within minutes [8], hours [9], or days [10,11,12] of the injection. The pathophysiology of angioedema is complex and can be triggered by one of several pathways, but is broadly classified as being either bradykinin-mediated or histamine-mediated [13]. Bradykinin is the central mediator responsible for triggering angioedema due to hereditary disease, acquired C1-inhibitor deficiency, and angiotensin-converting enzyme inhibitors (ACEis). It is not associated with urticaria, does not respond to corticosteroids or antihistamines, and incompletely responds to epinephrine [13,14]. In contrast, histamine release causes anaphylaxis and allergic angioedema, triggered by IgE-mediated mast cell degranulation, and is responsive to corticosteroids, antihistamines, and epinephrine [14]. Mast cells also release tryptase, a serum biomarker that elevates quickly and peaks at 1–2 h post-degranulation. In our patient, serum tryptase was normal when measured approximately nine hours after the semaglutide injection and, therefore, does not necessarily rule out a histamine-mediated reaction. In general, the pathophysiology of GLP-1 RA-mediated hypersensitivity reactions is incompletely characterized; however, extendin-based GLP-1 RA drugs (e.g., lixisenatide) more commonly cause histamine-mediated reactions than the human analogs (e.g., semaglutide, dulaglutide, and liraglutide) [15]. Based on our patient’s lack of urticaria and poor response to treatment with antihistamines, corticosteroids, and epinephrine, it is most likely that she exhibited a bradykinin-mediated reaction rather than histamine-mediated anaphylaxis or allergic angioedema. GLP-1 receptor signaling has also been shown to stimulate the release of Substance P [16], a known mediator of non-allergic angioedema that is also involved in ACEi-induced angioedema.

Diagnosis of GLP-1 RA-mediated angioedema is typically a clinical one based on presenting history, signs, and symptoms. Skin testing can be performed to investigate hypersensitivity to GLP-1 RAs [12], which was offered to our patient but was declined, and, therefore, limits our ability to definitively rule in a GLP-1 RA hypersensitivity reaction. However, skin testing can only detect histamine-mediated reactions that cause visible urticaria; it cannot rule in or rule out a bradykinin-mediated reaction. Given the close temporal relationship between the semaglutide injection and the onset of angioedema, the use of a GLP-1 RA was most likely the cause in this case. However, we cannot exclude other triggers of acquired angioedema, such as the use of valsartan, or synergism between semaglutide and valsartan. Based on the possible overlapping pathophysiology, patients developing angioedema from ACEis might also be at risk from GLP-1 RAs. We did not rule out acquired C1-inhibitor deficiency either, which would require screening for anti-C1q antibodies. The normal complement C4 level makes hereditary angioedema unlikely.

Recurrence of angioedema in patients taking ACEis can be seen in nearly half of patients even after discontinuation, although the vast majority (88%) occur within the first month [17]. Given our patient’s history of lisinopril-induced angioedema five years earlier, we cannot exclude the possibility of recurrent valsartan-induced angioedema. However, the five-year time lapse since her last exposure to lisinopril makes recurrent ACEi-induced angioedema less plausible. Moreover, recent studies have suggested that the risk of angioedema in patients taking angiotensin II receptor blockers (ARBs) may be similar to placebo, and ARBs are no longer contraindicated in patients with a history of ACEi-induced angioedema [18,19]. Additionally, up to 41% of patients diagnosed with angioedema due to ACEis/ARBs may be misdiagnosed [20]. Given the overlapping indications and patient populations taking both GLP-1 RAs and ACEis/ARBs, which may be as high as 30% [7], it is increasingly likely that patients presenting with angioedema will be taking both medications and require clinical adjudication and supportive testing.

The most common adverse effects of semaglutide are gastrointestinal symptoms, such as nausea, vomiting, diarrhea, constipation, and delayed gastric emptying. Rare but severe adverse reactions include acute pancreatitis and acute cholecystitis [21]. In contrast, the blood-sugar-lowering effect of semaglutide in obese, non-diabetic patients is modest, with a mean reduction in glycated hemoglobin of only 0.31 percentage points [1]. Hypoglycemia is infrequent, and in randomized controlled trials occurs at similar rates to placebo; however, there have been reports of hypoglycemia in post-marketing surveillance [22,23]. GLP-1 RAs may cause hypoglycemia by increasing insulin release in response to glucose, reducing glucagon secretion, and exhibiting a longer half-life than endogenous GLP-1 by overcoming degradation by dipeptidyl peptidase-4 [24]. Based on these mechanisms, hypoglycemia from semaglutide in our patient on hospital days 5–7 is possible, particularly after taking a full first dose. Another possible contributing factor may have been depletion of glycogen stores after receiving multiple doses of epinephrine. Her development of nausea and vomiting over the same time frame also supports an adverse reaction to semaglutide as the underlying cause.

Our case highlights the ease with which dosing errors can be made by patients self-administering compounded GLP-1 RA drugs, some of which have led to hospitalization [6,25]. While our patient injected a ten-times higher dose than intended on the first injection (2 mg), it was within the limits of the maximum weekly dose approved for weight loss (up to 2.4 mg). Therefore, while the inadvertently higher dose on the first injection is clinically relevant, the dose itself seems less likely to be the driver of our patient’s reaction. Adverse events from compounded GLP-1 RAs appear to exceed those of the FDA-approved formulations [26], and vulnerable patients who lack experience with units of measure are particularly at risk [25]. In addition, our case illustrates the unknowns of how direct-to-consumer online and compounding pharmacies develop their formulations. We do not know the complete list of ingredients in the compounded product, and it is possible that our patient’s reaction was due to an inactive ingredient, excipient, contaminant, or impurity in the formulation, rather than to the semaglutide itself. Despite recent issuance from the FDA [27], GLP-1 RA dosing errors and adverse reactions will likely continue, as patients continue to purchase compounded, non-FDA-approved semaglutide [28].

## 4. Conclusions

We report a case of severe, life-threatening angioedema that developed several hours after her first injection of semaglutide. This adverse drug reaction was associated with a dosing error of unclear clinical relevance. The patient’s history of lisinopril-induced angioedema five years earlier and concurrent use of valsartan raised uncertainty over whether she was presenting with recurrent ACEi-associated angioedema; however, the timing of the lisinopril and the literature do not support that diagnosis. Clinicians should be aware that GLP-1 RA use is associated with rare hypersensitivity reactions, including angioedema, and should inquire about dosing in patients presenting with potential complications or adverse reactions. Because the cross-reactivity between GLP-1 RA drugs is unknown, initiation of another drug within the class after a bout of angioedema should be done cautiously.

## Data Availability

The data presented in this study are available upon request.
